# Determinants and rates of retention in HIV care among adolescents receiving antiretroviral therapy in Windhoek, Namibia: a baseline cohort analysis

**DOI:** 10.1186/s12889-023-15356-w

**Published:** 2023-03-08

**Authors:** Farai K. Munyayi, Brian E. van Wyk

**Affiliations:** School of Public Health, University of the Western Cape, Cape Town, South Africa

**Keywords:** Adolescents, HIV, Retention in care, Antiretroviral therapy, Lost to follow up

## Abstract

**Background:**

Long-term engagement in HIV care is essential to achieving and maintaining viral suppression. Adolescents living with HIV (ALHIV) experience many barriers to remaining engaged in care and treatment programs. Higher attrition among adolescents compared to adults remains a huge concern due to unique psychosocial and health systems challenges adolescents face, and recently the COVID-19 pandemic effects. We report on determinants and rates of retention in care in adolescents aged 10–19 years enrolled on antiretroviral therapy (ART) in Windhoek, Namibia.

**Methods:**

A retrospective cohort analysis of routine clinical data of 695 adolescents aged 10–19 years enrolled for ART at 13 Windhoek district public healthcare facilities, between January 2019 and December 2021 was conducted. Anonymized patient data were extracted from an electronic database and registers. Bivariate and Cox proportional hazards analysis were performed to determine factors associated with retention in care among ALHIV at 6, 12, 18, 24 and 36 months. Retention in care trends were also described using the Kaplan-Meier survival analysis.

**Results:**

The retention in care rates at 6, 12, 18, 24 and 36 months were 97.7%, 94.1%, 92.4%, 90.2%, and 84.6%, respectively. Our study population had predominantly treatment-experienced adolescents, who initiated ART between birth and 9 years (73.5%), were on treatment for > 24 months (85.0%), and on first-line ART (93.1%). After controlling for confounders, the risk of dropping out of care was increased for older adolescents aged 15–19 years (aHR = 1.964, 95% CI 1.033–3.735); adolescents on switched ART regimens (Second line + Third line regimen) (aHR = 4.024, 95% CI 2.021–8.012); adolescents who initiated ART at 15–19 years (aHR = 2.179, 95%CI 1.100-4.316); and male adolescents receiving ART at a PHC clinic (aHR = 4.322, 1.332–14.024). Conversely, the risk of ALHIV dropping out of care decreased for adolescents whose TB screen results were negative (aHR = 0.215, 95% CI 0.095–0.489).

**Conclusion:**

Retention in care rates among ALHIV in Windhoek do not meet the UNAIDS revised target of 95%. Gender-specific interventions are needed to keep male and older adolescents motivated and engaged in long-term care, and to promote adherence amongst those adolescents who were initiated on ART in late adolescence (15–19 years).

## Introduction

In 2020, about 1.75 million [1.16 million–2.3 million] adolescents were estimated to be living with HIV, which reflects an increasing population of adolescents living with HIV (ALHIV) across the world compared to previous years [1]. Adolescence is described by the World Health Organization (WHO) as the life phase after childhood and before reaching adulthood, specifically between the ages of 10 and 19 years old [2]. Estimates for 2020 alone indicate that about 150,000 adolescents (10–19 years) were newly infected with HIV worldwide [1]. The newly infected adolescents add to the already growing sub-population of ALHIV considering the number of vertically infected children surviving into adolescence [3]. While children continue to be underserved by most health systems in terms of access to appropriate HIV services, the COVID-19 pandemic exacerbated the lack of access, with several restrictive measures being implemented to mitigate the spread of the SARS-CoV-2 virus [4]. If the current trajectory continues, not only would the efforts to achieve epidemic control remain a far-to-reach goal, but the gains in the pre-COVID-19 period may be reversed. Increased focused and concentrated efforts are needed on ALHIV to get back on track and make progress towards achieving HIV epidemic control [1].

Global efforts to achieve HIV epidemic control include commitments by national HIV programs to develop and implement strategies to meet the recently revised UNAIDS 95-95-95 targets, which aim to ensure that 95% of all PLHIV know their status, 95% of all HIV positive individuals are initiated and retained on antiretroviral therapy (ART), and 95% of all individuals on ART achieving viral suppression [5]. Namibia has adopted strategies such as the treat-all approach, also known as the test-and-treat strategy, whereby all individuals diagnosed HIV positive are immediately initiated on ART [6]. Although the program has achieved huge successes on same-day ART initiations, concerns remain on the retention of these newly diagnosed and newly initiated individuals on ART. More so, retention in care remains a great concern among ALHIV, who face unique challenges and continue to be underserved by most health systems in sub-Saharan Africa. In most sub-Saharan Africa countries, public health facilities are ill-equipped to give guidance and support for ALHIV to remain engaged in care as services are arranged to serve younger children in pediatric HIV clinics or adults, in adult care clinics [7]. A recent study on viral non-suppression in adolescents enrolled in ART services in Windhoek reported that 16.4% of unsuppressed adolescents were not engaged in HIV care [8].

The relationship between retention in HIV care and viral suppression has been well established through several studies. A study conducted in South Africa found that 73% of all adolescents and young adults who were included in the study had the composite outcome of being retained in care and virally suppressed within the previous 12 months, with a correlation of even higher retention and viral suppression rates of 97% and 91% respectively, among adolescents attending a dedicated adolescents clinic [9]. A youth friendly service in London (UK) found higher rates (81%) of viral suppression at a threshold of < 200 copies RNA/ml in adolescents who were retained in care whilst in the US viral suppression was also high (> 70%) among those retained in care [10, 11]. In Nigeria, an analysis of continuity of care and viral suppression among adolescents and young adults reported that males were less likely to be retained in care and were subsequently less likely to be virally suppressed [12]. Retaining adolescents in HIV care services ensures continuous access to medications and is a critical precursor to better adherence to ART, and achieving and maintaining viral suppression [13]. Interventions to improve treatment outcomes in ALHIV should therefore address barriers to retention in care, adherence to ART and achievement of viral suppression. The Fast-Track Cities Initiative, which is a multi-country initiative launched during the 2014 World AIDS day commemorations, targets cities as essential components playing a critical role in the HIV response [14].

Windhoek is one of the cities that joined the Fast-Track Cities Initiative, which highlights optimization of HIV service delivery especially for adolescents, and promoting long-term engagement and enhancing retention in HIV care [15]. Windhoek is the capital city of Namibia and has approximately 431 000 inhabitants. The 2017 NAMPHIA study reported that Namibia had achieved an estimated 86-96%-91% of the 90-90-90 UNAIDS targets, and had an overall 4.0% prevalence of HIV among young people aged 15 to 24 years, and 1.7% among young adolescents aged 10–14 years [16]. Recent modelled spectrum estimates have reported that Namibia has reached 94 -97% -93% of the revised 95-95-95 UNAIDS and WHO targets. However, these overall estimates regrettably mask the gaps that may exist within different sub-populations. Routine data to specifically monitor HIV treatment outcomes and performance indicators among adolescents (10–19 years) is often not available because of routine disaggregation of reported data with definitions of children as < 15 years and adults as 15 years and above [17, 18]. However, a study conducted in 2020 that evaluated a teen club intervention at a specialized pediatric ART clinic in Windhoek found an overall retention in HIV care rate of approximately 90.1% at 24 months among all adolescents aged 10–19 years enrolled for ART at the clinic [19]. Besides the specialized pediatric ART clinic at one of the referral hospitals in Windhoek, the public healthcare infrastructure where ALHIV receive ART services includes another major referral hospital, two healthcare centres, and nine primary healthcare clinics.

Retention in HIV care is defined as a continuous engagement in a package of prevention services, treatment, care and support services, from the point of diagnosis, and the enrolled individual routinely attends these services as needed [20]. A systematic review conducted in 2017 found no retention in HIV care studies or interventions exclusively targeting adolescents aged 10–19 years, although one study reported no effect on retention rates in youths aged 15–25 years who were exposed to a youth-friendly clinic compared to those in a family-oriented clinic [13]. However, the study reported findings of significant associations between retention in HIV care among adults and decentralization, down-referral of stable patients, task-shifting of services, and differentiated care service delivery models [13]. Whilst a study in Uganda found a greater risk of non-retention in care in older adolescents compared to younger adolescents group (10–14 years), an evaluation of the teen club intervention in Namibia also found poorer retention rates among the older adolescents aged 15–19 years [7, 19]. In addition, pediatric HIV disclosure (the process of informing a child/adolescent of their HIV status) is reportedly associated with retention in care among adolescents, and the WHO recommends early disclosure of HIV status as a measure to improve treatment outcomes in adolescents [21]. The need for designing and implementing new innovations targeting better retention in care in ALHIV becomes more pronounced as we strive to close the remaining gap towards reaching the 95-95-95 targets and achieving and maintaining epidemic control.

## Methods

### Study design and participants

We conducted a quantitative, retrospective cohort analysis of routine demographic and clinical data of ALHIV aged 10–19 years enrolled at 13 public healthcare facilities providing ART in the Windhoek district from January 2019 to December 2021. Individual anonymized patient-level data was extracted from the standard routine ART database used at all the public healthcare facilities in Namibia, the electronic Patient Monitoring System (*e*PMS). The *e*PMS is an electronic health information system used in public health facilities in Namibia to routinely monitor and manage clients on ART across the country. Data clerks based at each facility are responsible for entering patient data recorded by clinicians in patient files, the Patient Care Booklets (PCB), into the *e*PMS. The study data was extracted on the 25th of February 2022, consisting of anonymized data identified through unique patient identifiers. The data extracted included sociodemographic variables, and the related clinical and treatment outcomes data collected during routine clinic visits for clinical management and monitoring of all patients receiving ART. Patient Care Booklets for participants with incomplete records in the *e*PMS were retrieved, and any available unentered information was updated in the electronic system.

### Data collection

The primary outcome of this study was retention in HIV care. Longitudinal data were collected from routine clinic visits of all adolescents enrolled for ART in the Windhoek district. We considered ‘point’ retention at 6 months post-ART initiation, which can be defined as alive or the presence of individuals in ART services six months after initiating ART or any time thereafter [22]. We considered 36-month retention in care from January 2019 to December 2021, using six monthly intervals. The programmatic definition using the six months ‘point’ retention was preferred to align with recent guidance on multi-month prescription and multi-month dispensing of ART in Namibia [23]. The predictor variables extracted from the electronic database include age, sex, WHO stage at ART initiation, age at ART initiation, ART regimen at initiation, current ART regimen, duration on ART, TB screening results, HIV disclosure status, HIV viral load results and healthcare facility level.

### Data analysis

The extracted data from the ePMS was matched and merged with the patient data obtained from the PCBs using the unique ART numbers allocated (unique patient identifiers) at each ART clinic. A Microsoft Excel (Microsoft Corporation, Washington, DC, USA) spreadsheet was created from the merged data. The extracted anonymized data was saved into a password-protected excel file to prevent any unauthorized access or alterations of the data. The merged complete excel spreadsheet was imported into the SPSS statistical software (IBM SPSS version 28, IBM Corp. USA) for analysis. Descriptive statistics were carried out to describe the demographic and clinical characteristics of the adolescent participants included in the study, at baseline and during the following 36-month period. Bivariate analysis was executed utilizing the Chi-square test to determine the association, and the significance thereof, between retention in care and the demographic and clinical variables (age, sex, duration on ART, age at ART initiation, HIV disclosure status, WHO stage at ART initiation, current ART regimen, ART regimen at initiation, TB screening results, viral suppression status and healthcare facility level). Comparisons were performed between retention in care and demographic and clinical parameters at 6, 12, 18, 24 and 36 months post initiation on ART. Fisher’s exact test was used as an alternative to the Chi-square test in instances of sparse data (< 5 in any cell).

The Cox regression (Cox Proportional Hazards model) analysis was performed to adjust for potential confounders and interactions, to determine predictors for retention in care. The Cox proportional hazard model utilized the backward stepwise analysis, with the initial model inclusive of all candidate variables. The least significant variable was subsequently removed at each iteration until none of the nonsignificant variables remained. Variables were removed from the model at a set significance level of p < 0.05. The Complete Case Analysis (CCA) was used as less than 5% of the cases had missing data on all variables in the main analysis. Survival analysis was assessed with “Patient retention status at the end of the period” as the outcome of interest. A comparative survival analysis for the age and sex of the study participants using Kaplan-Meier survival curves was conducted [24]. Using Cox regression, factors influencing retention in care at months 6, 12, 18, 24 and 36 were established. Both the unadjusted and adjusted hazard ratios and their p-values were computed.

### Ethics approval

The ethical approval was obtained from the Biomedical Research Ethics Committee at the University of the Western Cape (ref. no. BM21/5/7) in Cape Town, South Africa, and the Namibia Ministry of Health and Social Services (MoHSS) Research Management Committee (ref. no. 17/3/3/FKM). Permission was also obtained from the MoHSS to get access to the Patient Care Booklets (PCBs) and the electronic Patient Monitoring System (*e*PMS). No personal identifying information such as patient names, surnames or identity numbers, were extracted from the electronic database or from the review of participants’ PCBs, to ensure respect for the privacy and dignity of the participants and confidentiality of participants’ information. Informed consent was waivered by both ethics committees for this phase of the study, therefore no informed consent process was required since data was extracted from routine databases. The study was carried out in compliance with the Helsinki guidelines declaration of 1964 and its subsequent amendments.

## Results

Table 1 shows the demographic characteristics and clinical history of 695 ALHIV enrolled in the ART programme in Windhoek District, Khomas region, Namibia in 2019. The median duration on ART was 8 years (IQR 6–12 years). Data was censored as the number of adolescents retained in care decreased at every six-monthly time points used in this analysis. Retention in care rates at 6, 12, 18, 24 and 36 months were 97.7%, 94.1%, 92.4%, 90.2%, and 84.6%, respectively. Most of the adolescents had initiated ART from birth to 9 years (73.5%). Compared to adolescents who initiated ART between 10 and 14, and 15–19 years of age, adolescents whose age at ART initiation was 0–9 years were more likely to be retained in care at 6, 12, 18, 24 and 36 months (p < 0.001). The distribution of the adolescents with regard to the current ART regimen was, first line treatment (93.1%), second line treatment (6.6%) and third line treatment (0.3%). Adolescents on first line treatment were more likely to be retained in care compared to their counterparts on second line treatment at all time points (p = 0.017). Most of the adolescents had been on ART for over 24 months (85.0%), with 11.5% having been on ART from 0 to 6 months. Very few adolescents had been on ART for 7–12 months (0.6%), 13–18 months (1.4%) and 19–24 months (1.4%), and adolescents who had been on ART for over 24 months were more likely to be retained in care compared to those who had been on ART from 0 to 6 months (p < 0.001).

The majority of the participants had negative TB screen results (94.1%), and adolescents with positive TB screen results were less likely to be retained in care compared to those who had negative TB screen results (p = 0.002). Regarding facility level, most of the adolescents were being treated at hospital settings (49.4%) and as compared to those attending Health Centres and Primary Healthcare (PHC) clinics, adolescents being treated at hospitals were more likely to be retained in care (p < 0.001). Almost all the adolescents had disclosed their HIV status (99.0%). Overall, those who disclosed their HIV status were more likely to be retained in care, with longer duration on ART, compared to their counterparts who had not disclosed their status, at 18, 24 and 36 months (p = 0.013).

With regard to viral load suppression, 73.0% of the adolescents had fully suppressed viral loads; 15.7% were suppressed with low-level viraemia (40 -1000 copies/ml); and the remaining 11.3% had unsuppressed viral loads. Adolescents who were suppressed were more likely to be retained in care compared to those in the other categories at all time points. However, these differentials were not significant (p = 0.737).

**Table 1:** Retention in HIV care status by demographic and clinical characteristics of the adolescents (10–19 years) on ART in Windhoek District, Khomas Region, Namibia 2019–2021 (N = 695)

		Retained in care (Months)				
Characteristic	Total	6	12	18	24	36
Overall	695 (%)	679 (97.7)	654 (94.1)	642 (92.4)	627 (90.2)	588 (84.6)
**Sex**						
Female	394 (56.7)	380(96.4)	361(91.6)	353(89.6)	346 (87.8)	325 (82.5)
Male	301 (43.3)	299 (99.3)	293 (97.3)	289 (96.0)	281 (93.4)	263 (87.4)
**Age group (years)**						
10–14	264 (38.0)	262 (99.2)	261 (98.9)	257 (97.3)	254(96.2)	240 (90.9)
15–19	431 (62.0)	417 (96.8)	393 (91.2)	385 (89.3)	373 (86.5)	348 (80.7)
**Age at ART initiation (years)**						
0–9	511 (73.5)	506 (99.0)	501 (98.0)	493(96.5)	487(95.3)	457 (89.4)
10–14	96 (13.8)	92 (95.8)	89 (92.7)	89 (92.7)	83 (86.5)	78 (81.3)
15–19	88 (12.7)	81(92.0)	64 (72.7)	60(68.2)	57 (64.8)	53 (60.2)
**Duration on ART (months)**						
0–6	80 (11.5)	73 (91.3)	56 (70.0)	52 (65.0)	51 (63.7)	47 (58.8)
7–12	4 (0.6)	3 (75.0)	3 (75.0)	3 (75.0)	2 (50.0)	2 (50.0)
13–18	10 (1.4)	10 (100.0)	10 (100.0)	10(100.0)	10 (100.0)	10(100.0)
19–24	10 (1.4)	10 (100.0)	9 (90.0)	9(90.0)	8 (80.0)	8 (80.0)
> 24	591 (85.0)	583 (98.6)	576 (97.5)	568 (96.1)	556 (94.1)	521 (88.2)
**ART regimen at initiation**						
NNRTI based	636 (91.5)	620(97.5)	600 (94.3)	590 (92.8)	575(90.4)	540 (84.9)
PI based	48 (6.9)	48(100.0)	45(93.8)	45(93.8)	45(93.8)	41 (85.4)
DTG based	11 (1.6)	11 (100.0)	9(81.8)	7(63.6)	7(63.6)	7 (63.6)
**Current ART regimen**						
First line	647 (93.1)	634(98.0)	612(94.6)	603(93.2)	590(91.2)	554 (85.6)
Second line	46 (6.6)	43(93.5)	40(87.0)	37 (80.4)	35 (76.1)	33 (71.7)
Third line	2 (0.3)	2 (100.0)	2(100.0)	2(100.0)	2(100.0)	1 (50.0)
**HIV Disclosure status**						
Disclosed	667 (99.0)	652(97.8)	627(94.0)	618(92.7)	605 (90.7)	567 (85.0)
Not disclosed	7 (1.0)	7 (100.0)	7(100.0)	4(57.1)	3(42.9)	3 (42.9)
Missing	21 (3.0)					
**TB Screen results**						
Incomplete	23 (3.3)	20(87.0)	19 (82.6)	19(82.6)	16(69.6)	14 (60.9)
Negative	654 (94.1)	642(98.2)	621(95.0)	609(93.1)	597(91.3)	561 (85.8)
Positive	18 (2.6)	17(94.4)	14(77.8)	14(77.8)	14(77.8)	13 (72.2)
**WHO clinical stage at ART initiation**						
1	378 (67.3)	367(97.1)	353(93.4)	345(91.3)	334(88.4)	315 (83.3)
2	100 (17.8)	98(98.0)	92(92.0)	90(90.0)	87(87.0)	80 (80.0)
3	65 (11.6)	62(95.4)	59(90.8)	58(89.2)	57(87.7)	52 (80.0)
4	19 (3.4)	19(100.0)	19(100.0)	19(100.0)	19(100.0)	16 (84.2)
Missing	133 (19.1)					
**Facility level**						
Health centre	175 (25.2)	171(97.7)	163(93.1)	160(91.4)	155(88.6)	146 (83.4)
Hospital	343 (49.4)	341(99.4)	341(99.4)	336(98.0)	332(96.8)	313 (91.3)
PHC clinic	177 (25.5)	167(94.4)	150(84.7)	146(82.5)	140(79.1)	129 (72.9)
**Viral load suppression (copies/ml)**						
Fully suppressed (< 40)	506 (73.0)	495 (97.8)	474 (93.7)	464 (91.7)	453 (89.5)	426 (84.2)
Suppressed (40-1000)	109 (15.7)	109 (100)	107 (98.2)	106 (97.2)	104 (95.4)	95 (87.2)
Not suppressed (> 1000)	78 (11.3)	73 (93.6)	71 (91.0)	70 (89.7)	69 (88.5)	66 (84.6)
Missing	2 (0.3)					

**Fig. 1:**
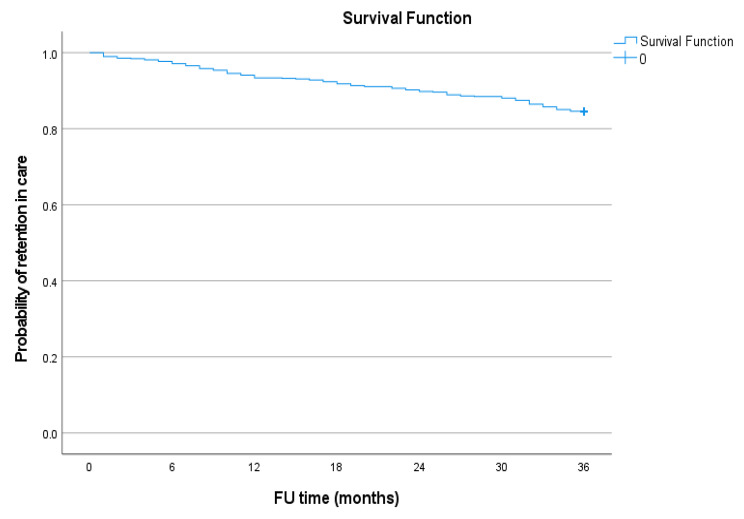
Kaplan-Meier survival estimates for retention in care among adolescents over a 3-year period

**Fig. 2:**
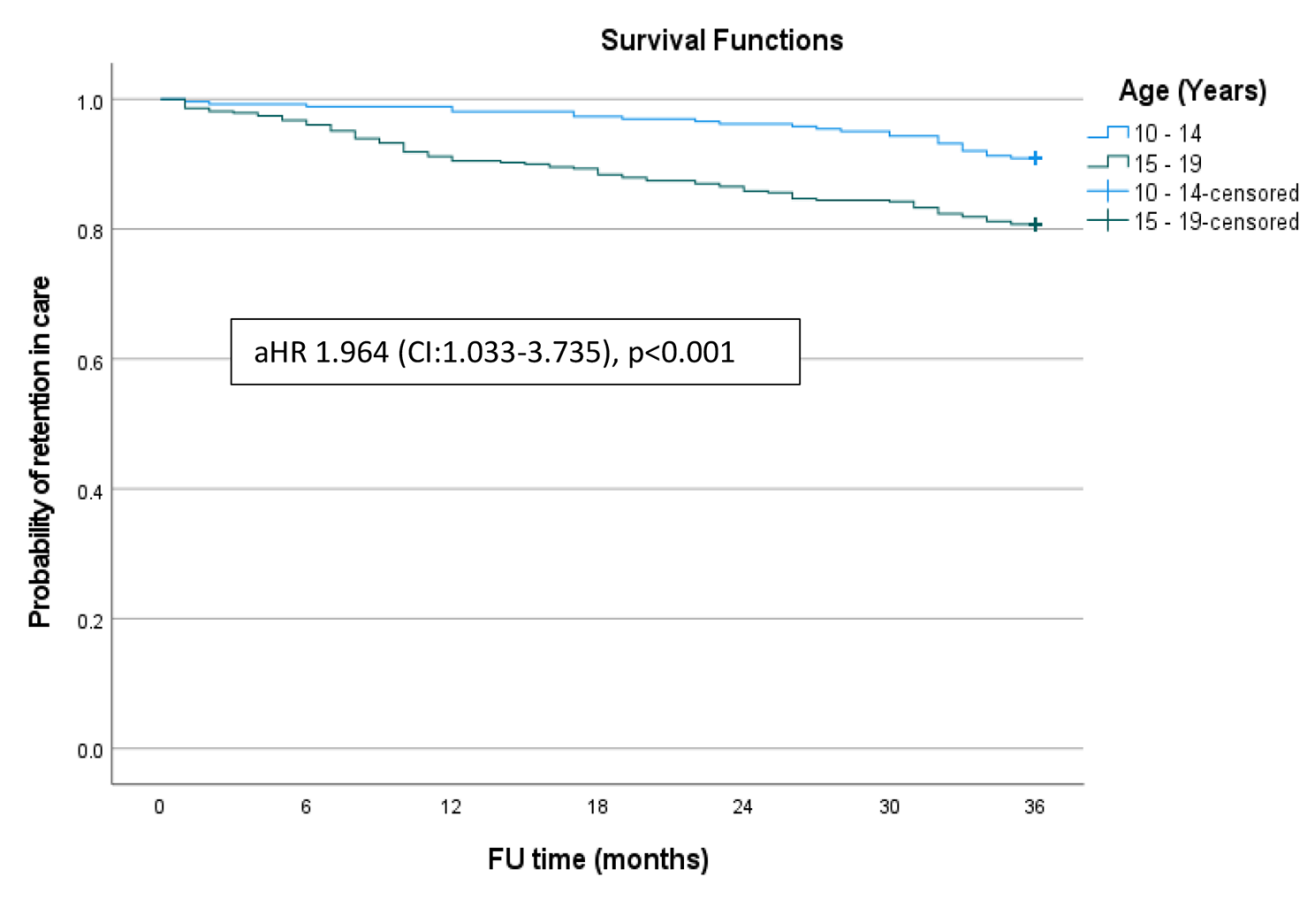
Kaplan Meier survival curves for retention status by age

**Fig. 3:**
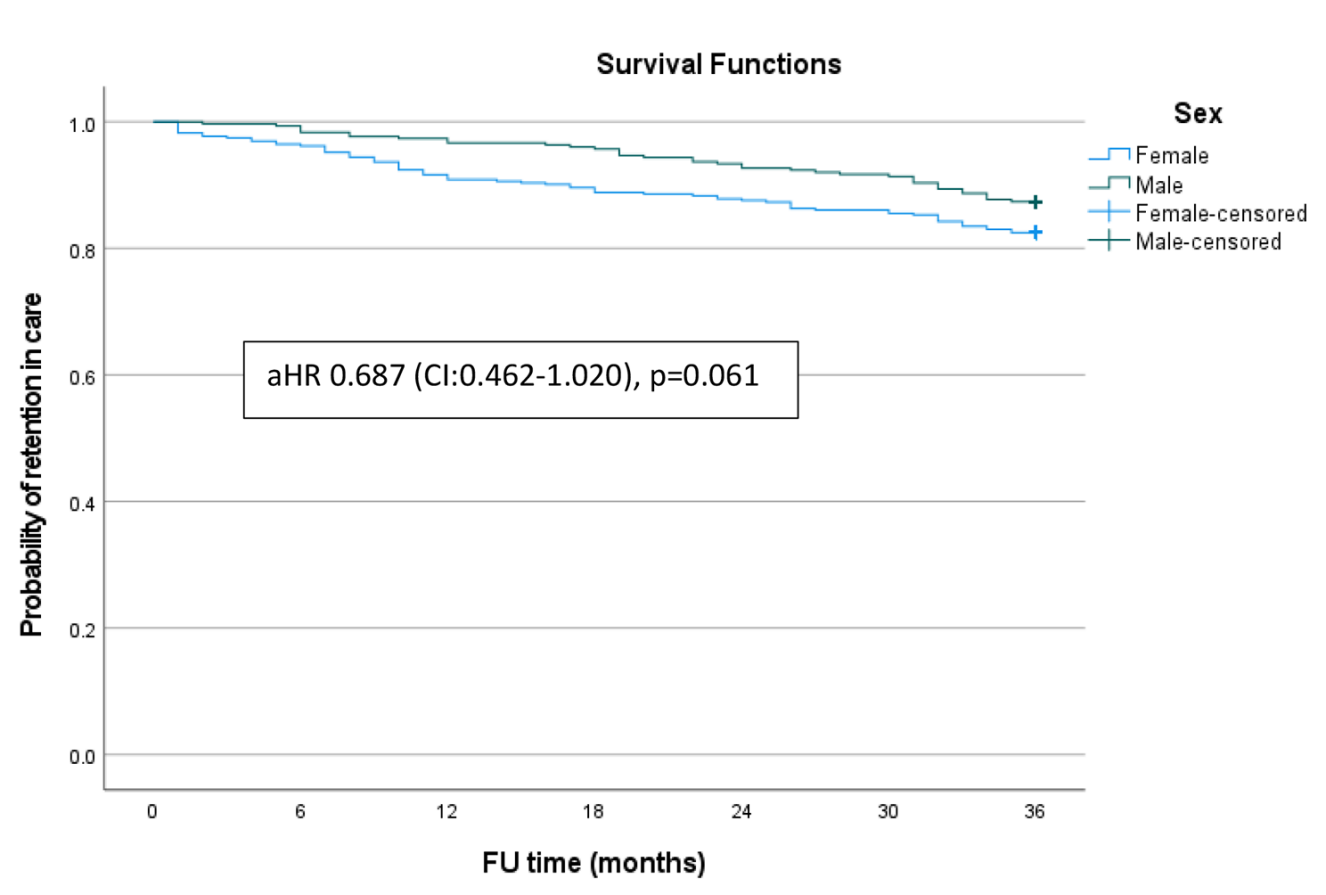
Kaplan Meier survival curves for retention status by sex

Figure 1 shows the Kaplan-Meier survival estimate for retention in care among ALHIV enrolled in the ART programme in Windhoek District, Khomas region. Figure 2 shows the Kaplan-Meier survival curves for retention in care by age of the adolescents. There were significant differences in retention in care between the 10–14 and 15–19 age groups (Log Rank = 13.606, p < 0.001). Compared to their 15 to 19-year-old counterparts, the younger adolescents were more likely to be retained in care at 6 months (99.2% versus 96.8%), 18 months (98.9% versus 91.2%), and 36 months (90.9% versus 80.7%). Figure 3 shows the Kaplan-Meier survival curve for retention status by sex of the adolescents over the study period. There were no significant differences in retention rates between male and female participants (Log Rank = 3.522, p = 0.061).

### Factors associated with retention in HIV care among adolescents living with HIV

Table 2 shows the Cox proportional hazard analysis to understand risk factors for retention in care among adolescents. After controlling for the effect of cofounders, retention on ART was significantly influenced by age, current ART regimen, and TB screen results. The risk of dropping out of ART care increased for adolescents aged 15–19 years (aHR = 1.964, 95% CI 1.033–3.735); adolescents on switched ART regimens (Second line + Third line regimen) (aHR = 4.024, 95% CI 2.021–8.012); adolescents who initiated ART at 15–19 years (aHR = 2.179, 95%CI 1.100-4.316) years of age; and male adolescents receiving ART at a PHC clinic (aHR = 4.322, 1.332–14.024). Conversely, the risk of ALHIV dropping out of ART care decreased for adolescents whose TB screen results were negative (aHR = 0.215, 95% CI 0.095–0.489).

**Table 2:** Cox proportional hazard analysis to establish risk factors for retention in care among the adolescents

	Total (N = 695)		Females (N = 394)		Males (N = 301)	
	Crude HR (95%CI	aHR (95%CI)	Crude HR (95%CI)	aHR (95%CI)	Crude HR (95%CI)	aHR (95%CI)
**Sex**						
Female	1					
Male	0.687 (0.462–1.020)		-	-	-	-
**Age group (years)**						
10–14	1*	1*	1*		1	1*
15–19	2.291 (1.455–3.609)	1.964 (1.033–3.735)	3.048 (1.600-5.808)		1.486 (0.760–2.905)	4.499 (1.491–13.575)
**Age at ART initiation (years)**						
0–9	1*	1*	1*		1*	
10–14	1.896 (1.112–3.232)	1.322 (0.671–2.604)	1.962 (0.956–4.026)		1.837 (0.828–4.073)	
15–19	4.917 (3.211–7.530)	2.179 (1.100-4.316)	5.098 (3.055–8.506)		3.496 (1.338–9.134)	
**Duration on ART (Months)**						
<=24	1*		1*	1	1	
> 24	0.266 (0.178–0.396)		0.236 (0.147–0.380)	0.463 (0.211–1.017)	0.456 (0.191–1.091)	
**Current ART regimen**						
First line	1*	1*	1*	1*	1	
Switched (Second Line + Third line)	2.257 (1.287–3.595)	4.024 (2.021–8.012)	2.801 (1.433–5.478)	6.598 (2.939–14.816)	1.568(0.556–4.419)	
**TB Screen results**						
Incomplete	1*	1*	1	1*	1	1*
Negative	0.295 (0.149–0.585)	0.215 (0.095–0.489)	0.460 (0.167–1.265)	0.230 (0.080–0.665)	0.167 (0.065–0.429)	0.223 (0.063–0.794)
Positive	0.658 (0.221–1.965)	0.152 (0.028–0.822)	1.477 (0.396-5.500)	0.264 (0.046–1.509)	0.000 (0.000- )	0.000 (0.000- )
**WHO clinical stage at ART initiation**						
1/2	1		1	1*	1	
3/4	1.091 (0.0.639–1.862)		1.149 (0.601–2.196)	2.406 (0.971–4.312)	0.974 (0.377–2.517)	
**Facility level**						
Health centre	1*		1*		1*	1*
Hospital	0.494 (0.296–0.823)		0.488 (0.258–0.923)		0.547 (0.230–1.298)	1.382 (0.411–4.465)
PHC clinic	1.774 (1.119–2.813)		1.475 (0.841–2.588)		2.430 (1.083–5.451)	4.322 (1.332–14.024)

## Discussion

This paper reports on the retention in HIV care rates and associated factors for ALHIV aged 10–19 years enrolled on ART in the Windhoek district of Namibia over a three-year period. The retention in care rates fall short of the revised UNAIDS and WHO targets of achieving 95-95-95 goals for all populations, and interventions to improve retention in care rates, particularly among ALHIV, are urgently needed. The majority of adolescents included in our study were treatment experienced adolescents with a median duration on ART of 8 years and 73.5% of them having initiated ART at birth or by the age of 9 years. Although the retention rates at 6 months were within the 95% target, the rates subsequently fell far short of the target from one year to the three-year retention rates. Comparatively, the retention rates in our study were slightly higher than reported in similar settings, likewise declines in retention in care rates over time among ALHIV enrolled in care were reported in a recent study in Mpumalanga, South Africa, which found retention rates of 90.5%, 85.4%, 80.8% and 76.2% at 6, 12, 18 and 24 months respectively [25]. Even though another study conducted in Cape Town, South Africa reported much lower retention in care rates among ALHIV over a 24 months period, similar trends of sharp decline in retention rates were observed from 4, 12, and up to 24 months [26]. In our study retention rates were above 90% at 24 months, which correlates with high viral suppression rates up to 24 months among the included participants. However, with lower retention rates beyond the 2-year period, the viral suppression rates also fell by year three of the study. Overall, it is evident that the longer we can keep adolescents engaged in care the better their treatment outcomes, particularly maintaining suppressed viral loads.

Our study findings also show that adolescents who initiated ART from birth to within 9 years of age were more likely to be retained in care throughout the 36-month period. These are likely to be more treatment experienced and would have benefited more from caregiver support during their earlier years of taking their medication into adolescence. It is also likely that they may have benefited from a structured way of disclosure of their HIV status and smoothly transitioned into more independent adolescent care. Adolescents initiating ART at an older age are at higher risk of non-retention as some initiate ART when they have advanced HIV disease or immunodeficiency, and late diagnosis and initiation on ART have been found to be associated with poor treatment outcomes especially among older adolescents (15–19 years) [21, 23, 24][27]. In addition to late diagnoses and late initiation, evidence also suggests that older adolescents on ART are at higher risk of being lost to follow up as they are transitioned from paediatric or adolescent care to adult ART services [28].

Adolescents who were on a first line ART regimen had better retention rates than those who had been switched to a second line ART regimen in our study. Individuals on ART are switched to rescue regimens based on poor treatment outcomes on the given first line regimens, and this could be due to ineffective first line treatment regimens in the presence of resistance mutations, which could also be a consequence of poor adherence to ART. The relationship between adherence, viral suppression and retention in HIV care has been well documented, and a study conducted in Tanzania reported that poor adherence to a first line ART regimen was a predictor of poor adherence to a second line regimen, whilst another study in Uganda reported that ALHIV with a history of viral non-suppression were at higher risk of having high viral loads even following switching of ART regimen [18, 26][29]. This provides supporting evidence to our finding poorer retention in care rates among adolescents switched to a second line regimen. On the contrary, a study conducted in South Africa found adolescents on a second line ART regimen to have better retention rates than those on a first line regimen [25]. However, it is imperative to strengthen monitoring and follow up mechanisms for individuals who are switched to second line regimens to ensure they remain engaged in care for improved treatment outcomes, particularly achievement and maintenance of viral suppression.

Our study found higher attrition levels among adolescents who were on treatment for a shorter duration (0–6 months) compared to those who had been on treatment for longer periods exceeding 24 months. As discussed above, older adolescents aged between 15 and 19 years had highest rates of loss to follow up, with a significant number attending the clinic only once with no follow up visits documented. Namibia implements a test-and-treat or treat-all strategy whereby all individuals who are diagnosed HIV positive are immediately initiated on treatment on the same day or within 7 days of the diagnosis [6]. Further studies may be worthwhile to explore the retention in care rates among newly diagnosed HIV positive adolescents and adults, in light of what appears to be significant attrition among adolescents included in our study. Evidence from elsewhere suggests that same day ART start improves clinical outcomes including retention in care and viral suppression justifying the WHO recommendation of accelerated ART initiation [30], although some recent studies have found same day ART start to be a risk factor for non-retention in care, with higher rates of attrition and loss to follow-up among “test-and-treat” era patients [31]. On the other hand, a study in South Africa found no significant association between same day ART initiation and retention in care among ALHIV [25].

Our study showed that adolescents who had a positive TB screen result were more likely to be lost to follow up compared to adolescents who had a negative TB screen result. Similar findings have been reported in sub-Saharan Africa with a study in South Africa reporting that adolescents who were diagnosed with TB disease were more likely to not be retained in HIV care or die as compared to those who had a negative TB diagnosis [25]. Adolescents may not have attained adequate neurocognitive maturity and may have increased difficulties in processing the long term consequences of discontinuing lengthy treatment regimens, such as for TB/HIV co-infection [32]. In Botswana, higher rates of loss to follow up were reported especially among adolescents in TB/HIV coinfection settings [33].

The level of the healthcare system where adolescents are receiving their HIV care appears to be a determinant of retention in care in our study. Adolescents who are enrolled in hospital settings had better retention rates than the lower-level facilities. In Windhoek district, one of the main hospitals provides more specialized pediatric HIV care, separate from adult clinic, with dedicated staff and implementing adolescent specific interventions such teen clubs, Namibia Adolescents Treatment Supporters (NATS) and other adolescent-friendly services. A recent study of the teen club intervention at this facility found high retention rates of above 90% overall among all adolescents aged 10–19 years included in the study [19]. Evidence suggests that these interventions improve treatment outcomes among adolescents, and in addition, a recent systematic review found peer-led community-based Differentiated Service Delivery interventions such as NATS (locally adopted version of Community Adolescents Treatment Supporter – CATS), to be amongst the most promising interventions to improve outcomes among ALHIV [30, 31][34][35]. Namibia has been scaling up these interventions to lower-level facilities to improve retention in care, adherence to ART and viral suppression among adolescents. It is essential to investigate facility level and settings as differences in facility characteristics and service delivery models can account for variances or disparities in retention in care rates from one facility to another [12].

Adolescent HIV treatment and care services are complicated by psychosocial issues such as stigma, mental health needs, family issues, school and healthcare facility settings and service delivery challenges, as well as fear of HIV status disclosure [36]. Our study found better retention rates among adolescents who had their HIV status disclosed to them. Similar findings were also reported from the study on teen clubs conducted in Windhoek as well as a study in Kenya which reported rates of 64% retention among adolescents not disclosed to, 82% for those with partial disclosure, and 92% among those fully disclosed to [19, 32, 33][37][38]. Disclosure issues have been cited as one of the main barriers to retention in care among CALHIV and psychosocial support strategies such as disclosure support should be prioritized [27]. Finally, our study showed some level of resilience of the pediatric HIV care program in the face of the peak of the COVID-19 pandemic. Despite the COVID-19 mitigation measures that may have derailed progress in treatment outcomes among adolescents, the overall retention rates among ALHIV remained similar to the findings from the 2017 study conducted in Windhoek [19]. The effects of implementation of differentiated care models for adolescents during the COVID-19 pandemic such as teen clubs, NATS and multi-month dispensing, among others may need further investigations.

### Study limitations

This study is based on a retrospective cohort analysis which relied on patient data which is routinely collected by healthcare staff during clinic visits and interactions. Therefore, accuracy and completeness of the data that was available for extraction was heavily dependent on the robustness of the data collection and capturing of individual client records by the clinical staff and data clerks. Only the routinely collected variables could be extracted from the database and any other variables that may have been of interest, such as socioeconomic factors and other unaccounted for clinical parameters that are not routinely tracked by the healthcare system. For example, variables such as pregnancy status for female adolescents, alcohol use, and CD4 count were inconsistently and poorly tracked and captured and there were no sufficient entries to reliably include them in the analyses as covariates. Quantities of missing information on WHO clinical stage at ART initiation, HIV disclosure, viral load and TB screening results data could have potentially influenced the findings of this study. Despite the shortcomings of incomplete or missing data, analysis of routinely tracked and reported data for management of patients and managing health programs provides real-life observations into the HIV services delivery systems` strengths and areas of improvement for the particular study population, including information systems gaps as demonstrated by the incompleteness of the data. The sample of our study was also all-inclusive of all ALHIV in Windhoek district, reducing biases such as selection or sampling bias.

## Conclusion

Globally, adolescents enrolled in ART services are reportedly at much higher risk of attrition from HIV care compared to adults and children. Our study sought to determine the rates of retention in care and identify determinants or factors associated with retention in care among ALHIV. Retention in care rates in our study increasingly dropped over the 36-month period, concerningly to levels much lower than the UNAIDS target of 95%. Retention rates were significantly influenced by age, current ART regimen, age at ART initiation, TB screen results and level of healthcare facility. These findings provide preliminary data that can be a vehicle for further inquiry into age-specific and health systems factors associated with retention in care among heterogenous populations such as adolescents. Evidence-based, gender-sensitive interventions are needed to keep ALHIV motivated and engaged in long-term care, particularly, males and older adolescents in addition to those adolescents who were diagnosed and initiated on ART in late adolescence. Maintenance of high retention rates among ALHIV is key to the achievement of better overall treatment outcomes, including achievement and maintenance of viral suppression.

## Data Availability

Our study analyzed datasets belonging to the Ministry of Health and Social Services. The data is available from the corresponding author upon reasonable request.

## List of abbreviations

aHR: adjusted Hazard Ratio
ALHIV: Adolescents Living with HIV
ART: Antiretroviral therapy
CALHIV: Children and Adolescents Living with HIV
CATS: Community Adolescents Treatment Supporter
CI: Confidence Interval
DTG: Dolutegravir
ePMS: electronic Patient Monitoring System
HIV: Human Immunodeficiency Virus
IQR: Interquartile range
MoHSS: Ministry of Health and Social Services
NAMPHIA: Namibia Population-based HIV Impact Assessment
NATS: Namibia Adolescents Treatment Supporter
NNRTI: Non-nucleoside Reverse Transcriptase Inhibitors
PCB: Patient Care Booklet
PHC: Primary healthcare clinic
PI: Protease Inhibitors
PLHIV: People Living with HIV
TB: Tuberculosis
UNAIDS: The Joint United Nations Programme on HIV/AIDS
WHO: World Health Organization

## References

[1] UNICEF. HIV and AIDS in Adolescents - UNICEF Data [Internet]. UNICEF. 2021 [cited 2022 May 5]. Available from: https://data.unicef.org/topic/hiv-aids/

[2] WHO. Adolescent health [Internet]. World Health Organization. 2022 [cited 2022 May 5]. Available from: https://www.who.int/health-topics/adolescent-health#tab=tab_1

[3] Enane LA, Davies M-A, Leroy V, Edmonds A, Apondi E, Adedimeji A et al. Traversing the cascade: urgent research priorities for implementing the ‘treat all’ strategy for children and adolescents living with HIV in sub-Saharan Africa. J Virus Erad [Internet]. 2018;4:40–6. Available from: https://www.sciencedirect.com/science/article/pii/S2055664020303447 10.1016/S2055-6640(20)30344-7 PMC6248846 30515313

[4] UNAIDS. 2021 UNAIDS Global AIDS, Updates -. CONFRONTING INEQUALITIES Lessons for pandemic responses from 40 years of AIDS [Internet]. UNAIDS. 2021 [cited 2022 Jun 10]. Available from: https://www.unaids.org/sites/default/files/media_asset/2021-global-aids-update_en.pdf

[5] UNAIDS. Understanding Fast-Track: Accelerating Action to end the AIDS Epidemic by 2030.UNAIDS. 2020

[6] MoHSS. National Guidelines for Antiretroviral Therapy Sixth edition, August 2019, Ministry of Health and Social Services Directorate of Special Programmes. Windhoek:Republic of Namibia; 2019.

[7] Ssali L, Kalibala S, Birungi J, Egessa A, Wangisi J, Lyavala J et al. Retention of adolescents living with HIV in care, treatment, and support programs in Uganda. 2014 [cited 2018 Feb 27]; Available from: http://www.hivcore.org/Pubs/Uganda_AdolHAART_Rprt.pdf

[8] Munyayi FK, van Wyk B. Closing the HIV Treatment Gap for Adolescents in Windhoek, Namibia: A Retrospective Analysis of Predictors of Viral Non-Suppression. Int J Environ Res Public Heal 2022, Vol 19, Page 14710 [Internet]. 2022 Nov 9 [cited 2022 Nov 17];19(22):14710. Available from: https://www.mdpi.com/1660-4601/19/22/14710/htm 10.3390/ijerph192214710 PMC9690371 36429431

[9] Zanoni BC, Sibaya T, Cairns C, Lammert S, Haberer JE. Higher retention and viral suppression with adolescent-focused HIV clinic in South Africa. PLoS One [Internet]. 2017 [cited 2018 Oct 15];12(12):e0190260. Available from: http://www.ncbi.nlm.nih.gov/pubmed/29287088 10.1371/journal.pone.0190260 PMC5747481 29287088

[10] Foster C, Ayers S, McDonald S, Frize G, Chhabra S, Pasvol TJ et al. Clinical outcomes post transition to adult services in young adults with perinatally acquired HIV infection: Mortality, retention in care and viral suppression. AIDS [Internet]. 2020 Feb 1 [cited 2022 Nov 23];34(2):261–6. Available from: https://journals.lww.com/aidsonline/Fulltext/2020/02010/Clinical_outcomes_post_transition_to_adult.12.aspx 10.1097/QAD.0000000000002410 31651427

[11] Hall HI, Gray KM, Tang T, Li J, Shouse L, Mermin J. Retention in care of adults and adolescents living with HIV in 13 US areas. J Acquir Immune Defic Syndr [Internet]. 2012 May 1 [cited 2022 Nov 23];60(1):77–82. Available from: https://journals.lww.com/jaids/Fulltext/2012/05010/Retention_in_Care_of_Adults_and_Adolescents_Living.12.aspx 10.1097/QAI.0b013e318249fe90 22267016

[12] Badejo O, Noestlinger C, Jolayemi T, Adeola J, Okonkwo P, Van Belle S et al. Multilevel modelling and multiple group analysis of disparities in continuity of care and viral suppression among adolescents and youths living with HIV in Nigeria. BMJ Glob Heal [Internet]. 2020 Nov 1 [cited 2022 Nov 23];5(11):e003269. Available from: https://gh.bmj.com/content/5/11/e003269 10.1136/bmjgh-2020-003269 PMC7646327 33154102

[13] Murray KR, Dulli LS, Ridgeway K, Dal Santo L, De Mora DD, Olsen P et al. Improving retention in HIV care among adolescents and adults in low- and middle-income countries: A systematic review of the literature. PLoS One. 2017 Sep 1;12(9). 10.1371/journal.pone.0184879 PMC5621671 28961253

[14] UNAIDS. Fast-Track cities | UNAIDS [Internet]. 2022 [cited 2022 Sep 7]. Available from: https://www.unaids.org/en/cities

[15] Joint UNAIDS, Fast-Track UNAIDS-IAPAC. Cities Project — Outline | UNAIDS [Internet]. 2022 [cited 2022 Sep 6]. Available from: https://www.unaids.org/en/resources/documents/2022/FTC_outline

[16] NAMPHIA. Namibia Population-based HIV Impact Assessment (NAMPHIA) 2017 NAMPHIA 2017 COLLABORATING INSTITUTIONS The PHIA Project. 2019.

[17] Ferrand RA, Briggs D, Ferguson J, Penazzato M, Armstrong A, Macpherson P (2016). Viral suppression in adolescents on antiretroviral treatment: review of the literature and critical appraisal of methodological challenges. Trop Med Int Heal.

[18] Natukunda J, Kirabira P, Ong KIC, Shibanuma A, Jimba M. Virologic failure in HIV-positive adolescents with perfect adherence in Uganda: a cross-sectional study. Trop Med Health [Internet]. 2019 Jan 17 [cited 2022 Jun 9];47(1). Available from: https://pubmed.ncbi.nlm.nih.gov/30679930/10.1186/s41182-019-0135-z PMC6337787 30679930

[19] Munyayi FK, van Wyk B. The effects of teen clubs on retention in HIV care among adolescents in Windhoek, Namibia. South Afr J HIV Med [Internet]. 2020 [cited 2020 Aug 18];21(1). Available from: /pmc/articles/PMC7059250/?report=abstract 10.4102/sajhivmed.v21i1.1031 PMC7059250 32158557

[20] World Health Organization (2021). Consolidated guidelines on HIV prevention, testing, treatment, service delivery and monitoring : recommendations for a public health approach.

[21] Lencha B, Ameya G, Minda Z, Lamessa F, Darega J. Human immunodeficiency virus infection disclosure status to infected school aged children and associated factors in bale zone, Southeast Ethiopia: cross sectional study. BMC Pediatr [Internet]. 2018 Dec 15 [cited 2021 Mar 27];18(1):356. Available from: https://bmcpediatr.biomedcentral.com/articles/10.1186/s12887-018-1336-z 10.1186/s12887-018-1336-z PMC6236985 30442118

[22] Ahoua L, Arikawa S, Tiendrebeogo T, Lahuerta M, Aly D, Becquet R et al. Measuring retention in care for HIV-positive pregnant women in Prevention of Mother-to-Child Transmission of HIV (PMTCT) option B + programs: The Mozambique experience. BMC Public Health [Internet]. 2020 Mar 12 [cited 2023 Feb 4];20(1):1–10. Available from: https://bmcpublichealth.biomedcentral.com/articles/10.1186/s12889-020-8406-5 10.1186/s12889-020-8406-5 PMC7069209 32164601

[23] CDC (Centers for Disease Control and Prevention). Adapting HIV treatment: How PEPFAR-supported countries sustained HIV services during a pandemic [Internet]. Global HIV and TB. 2021 [cited 2023 Feb 4]. Available from: https://www.cdc.gov/globalhivtb/who-we-are/features/mmdfeaturestory.html

[24] Alemu GG, Nigussie ZM, Amlak BT, Achamyeleh AA. Survival time and predictors of death among HIV infected under five children after initiation of anti -retroviral therapy in West Amhara Referral Hospitals, Northwest Ethiopia. BMC Pediatr. 2022 Dec 1;22(1). 10.1186/s12887-022-03693-5 PMC9677693 36411424

[25] Okonji EF, Wyk B, Van, Mukumbang FC. Two-year retention in care for adolescents on antiretroviral therapy in Ehlanzeni district, South Africa: a baseline cohort analysis. AIDS Care [Internet]. 2022 [cited 2022 Nov 28]; Available from: https://pubmed.ncbi.nlm.nih.gov/35357245/10.1080/09540121.2022.2057409 35357245

[26] van Wyk B, Kriel E, Mukumbang F. Retention in care for adolescents who were newly initiated on antiretroviral therapy in the Cape Metropole in South Africa. South Afr J HIV Med [Internet]. 2020 Jul 22 [cited 2020 Aug 10];21(1):1077. Available from: https://sajhivmed.org.za/index.php/hivmed/article/view/1077/1929 10.4102/hivmed.v21i1.1077 PMC7433256 32832112

[27] Maskew M, Bor J, MacLeod W, Carmona S, Sherman GG, Fox MP. Adolescent HIV treatment in South Africa’s national HIV programme: a retrospective cohort study. Lancet HIV. 2019 Nov;1(11):e760–8. 10.1016/S2352-3018(19)30234-6 PMC7119220 31585836

[28] Meloni ST, Agaba P, Chang CA, Yiltok E, Oguche S, Ejeliogu E (2020). Longitudinal evaluation of adherence, retention, and transition patterns of adolescents living with HIV in Nigeria. PLoS ONE.

[29] Ramadhani HO, Bartlett JA, Thielman NM, Pence BW, Kimani SM, Maro VP et al. Association of First-Line and Second-Line Antiretroviral Therapy Adherence. Open Forum Infect Dis [Internet]. 2014 Sep 1 [cited 2022 Jul 29];1(2). Available from: /pmc/articles/PMC4281791/10.1093/ofid/ofu079 PMC4281791 25734147

[30] Ford N, Migone C, Calmy A, Kerschberger B, Kanters S, Nsanzimana S et al. Benefits and risks of rapid initiation of antiretroviral therapy. AIDS [Internet]. 2018 Jan 2 [cited 2022 Dec 3];32(1):17–23. Available from: https://pubmed.ncbi.nlm.nih.gov/29112073/10.1097/QAD.0000000000001671 PMC5732637 29112073

[31] Mayasi N, Situakibanza H, Mbula M, Longokolo M, Maes N, Bepouka B et al. Retention in care and predictors of attrition among HIV-infected patients who started antiretroviral therapy in Kinshasa, DRC, before and after the implementation of the ‘treat-all’ strategy. PLOS Glob Public Heal [Internet]. 2022 Mar 11 [cited 2022 Dec 3];2(3):e0000259. Available from: https://journals.plos.org/globalpublichealth/article?id=10.1371/journal.pgph.0000259 10.1371/journal.pgph.0000259 PMC10022330 36962315

[32] Mulongeni P, Hermans S, Caldwell J, Bekker LG, Wood R, Kaplan R. HIV prevalence and determinants of loss-to-follow-up in adolescents and young adults with tuberculosis in Cape Town. PLoS One [Internet]. 2019 Feb 1 [cited 2022 Nov 30];14(2). Available from: /pmc/articles/PMC6363173/10.1371/journal.pone.0210937 PMC6363173 30721239

[33] Enane LA, Lowenthal ED, Arscott-Mills T, Matlhare M, Smallcomb LS, Kgwaadira B et al. Loss to follow-up among adolescents with tuberculosis in Gaborone, Botswana. Int J Tuberc Lung Dis. 2016 Oct 1;20(10):1320–5. 10.5588/ijtld.16.0060 27725042

[34] Munyayi FK, van Wyk B, Mayman Y. Interventions to Improve Treatment Outcomes among Adolescents on Antiretroviral Therapy with Unsuppressed Viral Loads: A Systematic Review. Int J Environ Res Public Health [Internet]. 2022 Apr 1 [cited 2022 Dec 3];19(7):3940. Available from: https://www.mdpi.com/1660-4601/19/7/3940/htm 10.3390/ijerph19073940 PMC8997420 35409621

[35] Willis N, Milanzi A, Mawodzeke M, Dziwa C, Armstrong A, Yekeye I et al. Effectiveness of community adolescent treatment supporters (CATS) interventions in improving linkage and retention in care, adherence to ART and psychosocial well-being: a randomised trial among adolescents living with HIV in rural Zimbabwe. BMC Public Health [Internet]. 2019 Dec 28 [cited 2021 Mar 15];19(1):117. Available from: https://bmcpublichealth.biomedcentral.com/articles/10.1186/s12889-019-6447-4 10.1186/s12889-019-6447-4 PMC6348677 30691425

[36] Enane LA, Davies MA, Leroy V, Edmonds A, Apondi E, Adedimeji A et al. Traversing the cascade: urgent research priorities for implementing the ‘treat all’ strategy for children and adolescents living with HIV in sub-Saharan Africa. J Virus Erad [Internet]. 2018 [cited 2022 Nov 28];4(Suppl 2):40. Available from: /pmc/articles/PMC6248846/10.1016/S2055-6640(20)30344-7 PMC6248846 30515313

[37] Vreeman RC, Scanlon ML, Mwangi A, Turissini M, Ayaya SO, Tenge C et al. A cross-sectional study of disclosure of HIV status to children and adolescents in western Kenya. PLoS One [Internet]. 2014 [cited 2018 Oct 17];9(1):e86616. Available from: http://www.ncbi.nlm.nih.gov/pubmed/24475159 10.1371/journal.pone.0086616 PMC3903588 24475159

[38] Ouma J, Tembula G, McLigeyo A, Njogu F, Kiare J. Effects of Disclosure on Retention to HIV Care and Treatment among Adolescents aged 10–19 years in CHS-Supported Sites. In Kenya; 2016. p. 91873.

